# Study on the High-Efficiency Expression of Horseradish Peroxidase in *Pichia pastoris*

**DOI:** 10.3390/molecules30224374

**Published:** 2025-11-12

**Authors:** Yaping Wang, Yidan Jing, Weizhen Li, Yuqing Wang, Fei Li, Yimin Qiu, Ben Rao

**Affiliations:** 1 National Biopesticide Engineering Technology Research Center, Hubei Biopesticide Engineering Research Center, Hubei Academy of Agricultural Sciences, Biopesticide Branch of Hubei Innovation Centre of Agricultural Science and Technology, Wuhan 430064, China; 20110028@hubu.edu.cn (Y.W.); jyd2926128849@163.com (Y.J.); 18771521420@163.com (W.L.); m15572831569@163.com (Y.W.); feili@hbaas.ac.cn (F.L.); 2 State Key Laboratory of Biocatalysis and Enzyme, Engineering Hubei Collaborative Innovation Center for Green Transformation of Bio-Resources, Hubei Key Laboratory of Industrial Biotechnology, Biology Faculty of Hubei University, Hubei University, Wuhan 430062, China

**Keywords:** horseradish peroxidase, multiple copies, *Pichia pastoris*, co-expression of molecular chaperones, high-density fermentation

## Abstract

Horseradish peroxidase (HRP) is a heme-containing oxidoreductase with extensive applications in biotechnology, medical diagnostics, and environmental protection. In this study, *Pichia pastoris* was utilized to produce HRP. Successfully, expression strains with 1–5 copies of HRP-C were constructed, and the strain with the highest expression level and activity of HRP-C was obtained. Different molecular chaperones (PDI1, HAC1, BIP1) were selected, and co-expression was carried out through co-induction and separate induction methods. The results showed that the yield of HRP increased approximately 1.4 times with the assistance of PDI1 and HAC1 molecular chaperones in the 3-copy *Pichia pastoris* expression strain, with enzyme activities increasing by 1.2-fold and 1.3-fold, respectively. High-density fermentation of the recombinant strain transformed with BDM-PDI1-HRP-C-3C was carried out in a 50 L fermenter, and after methanol induction for 72 h, a target protein expression level of up to 200 mg/L was achieved. The enzyme activity reached 1796 U/mL, which is nearly three times higher than that of shake-flask fermentation and is the highest reported in the literature to date.

## 1. Introduction

Plant peroxidases (EC 1.11.1.7) are heme-containing enzymes consisting of an enzyme protein and an iron porphyrin complex. As one of the most widely used marker enzymes in the market, horseradish peroxidase is commonly used to label antibodies or other proteins for detection and quantification [1,2,3,4]. Most horseradish peroxidases are extracted and isolated from the horseradish plant and are used in biochemical assays to amplify weak signals and increase the signal of the target molecule being detected [1,2,3,4]. Therefore, enzyme activity is also an important detection index for this enzyme. Currently, commercial HRP preparations are usually a mixture of several isoenzymes isolated from horseradish, which greatly depends on the plant’s expression pattern [1,2,3,4]. To avoid the unpredictability caused by environmental influences, research on recombinant HRP has gained increasing attention. Currently, various organisms can serve as expression hosts for recombinant HRP. In 1992, HRP was secreted and expressed by insect Sf9 cell cultures and exhibited activity [1,2,3,4]. To produce HRP using Sf9 cells, hemin chloride needs to be added to the culture medium. After subsequent purification, up to 41.3 mg of active HRP was produced per liter of cell culture [5,6,7,8,9]. In 1994, HRP-C1A was overexpressed in tobacco, and it was found that the transformants grew 20% faster than wild-type plants [6,7,8,9]. In 1988, Chiswell and Ortlepp filed a patent application for the synthesis of a DNA sequence encoding HRP C [6,7,8,9,10]. The HRP gene was cloned into a vector and transformed into *Escherichia coli* for expression. In 1990, HRP expressed in *E. coli* was purified, and a refolding scheme was proposed [10,11]. Researchers have also used *Pichia pastoris* to study the C1A mutant, aiming to explore the potential of different yeast hosts in the production of highly glycosylated horseradish peroxidase and to provide an important experimental basis for enzyme property improvement [10,11]. *E. coli* is a commonly used expression host due to its simple and efficient expression system, which is easy to operate and scale up. In scientific research, the *E. coli* expression system is widely used in protein structure and function studies, providing an important experimental platform for elucidating the catalytic mechanism and biological activity of HRP [7,8,9,10]. Compared to *E. coli*, the *P. pastoris* expression system can provide more complex protein modifications and a correct protein folding environment, which is beneficial for producing HRP with high activity and stability [3,4,5,6,7,8,9]. HRP expressed in *Pichia pastoris* has advantages in producing active enzyme products, making it suitable for biotechnology, drug development, and clinical diagnostics. Additionally, as a eukaryotic expression system, *Pichia pastoris* can provide a protein synthesis environment closer to the natural situation, helping to better maintain the natural conformation and biological function of HRP [5,6,7,8,9,10,11]. Overall, selecting the appropriate expression system is of great significance for researchers and HRP production.

PDI1 (Protein Disulfide Isomerase) is a critical oxidoreductase that catalyzes the formation and isomerization of disulfide bonds in secretory proteins, ensuring proper folding and stability. It also acts as a chaperone to prevent misfolding and aggregates, particularly in the oxidative environment of the ER, where it collaborates with Ero1 to maintain redox balance [12]. HAC1, the homolog of mammalian XBP1, is a key transcription factor in the unfolded protein response (UPR). Upon ER stress, HAC1 mRNA is spliced by IRE1, activating genes encoding chaperones and foldases to alleviate protein-folding burdens [13]. BIP1 (Binding Immunoglobulin Protein) is the central ER chaperone that binds misfolded proteins, regulates UPR sensors, and facilitates proper folding by hydrolyzing ATP. It also mediates the retention of unfolded proteins in the ER for degradation via ERAD [14]. Their selection reflects complementary roles: PDI1 ensures oxidative folding, HAC1 orchestrates stress signaling, and BIP1 maintains protein quality control. Together, they optimize ER homeostasis and secretory capacity, making them essential targets for recombinant protein production in systems like *Pichia pastoris* [15].

This study utilized *Pichia pastoris* as the expression host, constructing an efficient HRP expression system through strategies such as gene dosage optimization and co-expression of molecular chaperones. Finally, by optimizing fermentation conditions and expression strategies, the study achieved high-efficiency expression and secretion of HRP in *Pichia pastoris*, ultimately achieving the goal of large-scale production of recombinant HRP. The developed recombinant HRP expression system can significantly reduce production costs, improve protein activity and yield, and help meet the demand for high-purity, high-activity HRP in medical diagnostics, environmental monitoring, and other fields. Further research on horseradish peroxidase can deepen the understanding of its structure and function, thereby exploring the applications of HRP and its variants in various fields.

## 2. Results and Discussion

### 2.1. Screening and Synthesis of Horseradish Peroxidase Gene

Based on literature reports and sequence alignment, horseradish and palm tree-derived peroxidases were screened, excluding those with low expression and activity. Four proteins were selected as candidates: 1QO4 [16], 1PA2 [17], 4USC [18], and HRP-C [9] (Genbank number: AAA72223.1).

**Figure 1:**
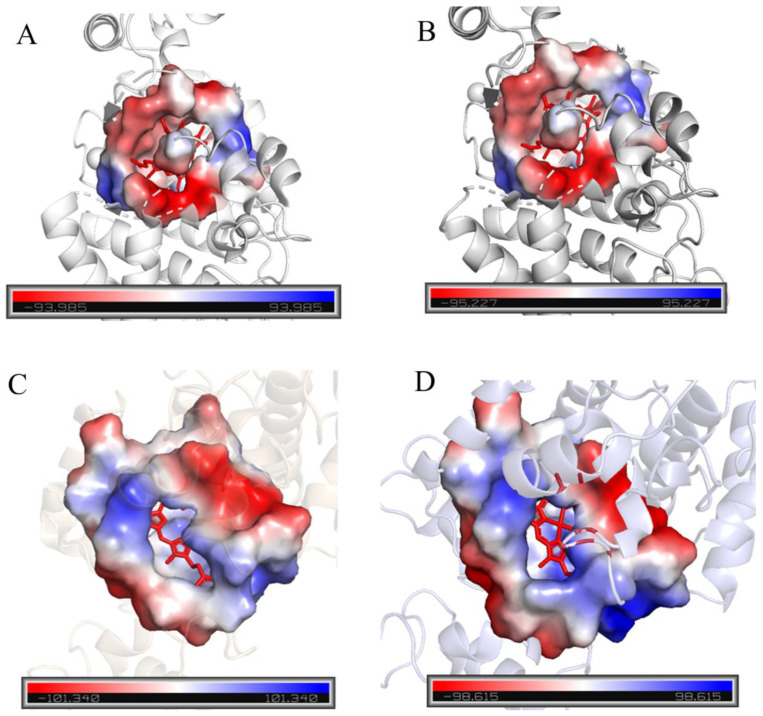
The hydrophobic pocket structure of HRP heme. (**A**): 1QO4; (**B**): 1PA; (**C**): 4USC; (**D**): HRP-C).

Current research indicates that the substrate of peroxidase is catalyzed by horseradish peroxidase in the heme pocket channel. Therefore, the size of the heme pocket may significantly affect peroxidase activity. To analyze the catalytic activity of candidate proteins, the heme pocket channels were analyzed in detail using SWISS-MODEL (https://swissmodel.expasy.org/) and PyMOL (https://www.pymol.org/). Figure 1A,B show that the hydrophobic heme pocket is blocked by several amino acid residues. Figure 1C,D show that HRP-C and two typical palm tree-derived peroxidases have relatively open hydrophobic pockets. Therefore, HRP-C, with the largest heme pocket, was selected as the target protein for subsequent experiments.

### 2.2. Expression and Identification of Multi-Copy HRP-C in Pichia pastoris

The constructed and verified multi-copy plasmids of HRP-C were electroporated into competent cells of *Pichia pastoris* GS115 in sequence. The selected positive clones were named GS115-BDM-HRP-C-2C, GS115-BDM-HRP-C-3C, GS115-BDM-HRP-C-4C, and GS115-BDM-HRP-C-5C, and stored at −80 °C.

**Figure 2:**
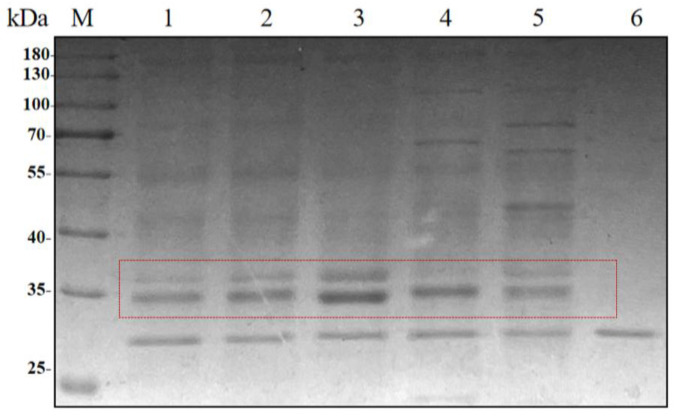
SDS-PAGE analysis of BDM-HRP-C fermentation in 1–5 copy shake flasks. M: PageRuler™ Prestained Protein Ladder; lanes 1–5: BDM-HRP-C fermentation results for one to five copy shake flasks; lane 6: Deglycosylation enzyme.

The recombinant strains with 1–5 copies of HRP-C were subjected to shake-flask induction. After 5 days of fermentation, the supernatant was co-incubated with recombinant EndoH at 37 °C for 2 h, followed by SDS-PAGE analysis (Figure 2). The results showed that the band width of the target protein increased with the copy number, reaching the highest value at three copies, while a decline was observed for four and five copies.

**Figure 3:**
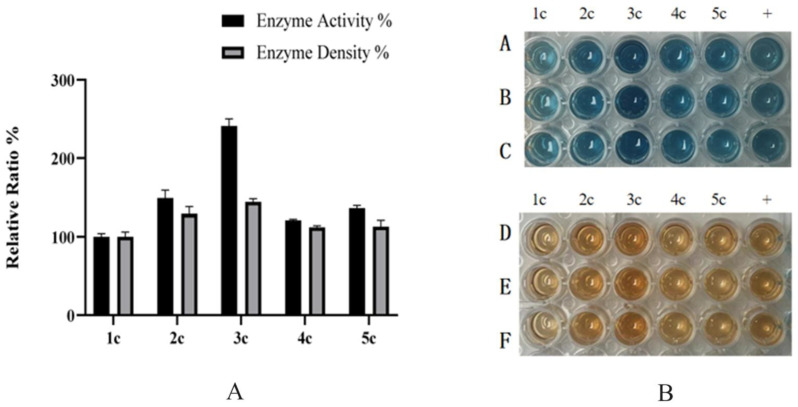
(**A**): Relative protein activity of shake flask supernatants from 1–5 copy expression strains; (**B**): A, B, C are the reactions with 5 μL enzyme solution added to 100 μL ABTS solution, reacted for 2 min, and then terminated with 2 M sulfuric acid; D, E, F are the reactions with 5 μL enzyme solution added to 100 μL Guaiacol solution, reacted for 2 min, and then terminated with 2 M sulfuric acid. Student’s *t*-test was used for statistical analysis, and a non-significant difference was detected between the control and experimental groups.

The expression levels of strains with 1–5 copies were measured using the Bradford quantitative assay in shake-flask fermentation. The results indicated that the expression levels of the 1–5 copy strains were 27 mg/L, 54 mg/L, 72 mg/L, 56 mg/L, and 57 mg/L, respectively (Figure 3A). Activity assays revealed that the three-copy HRP-C exhibited three-fold higher activity than the single-copy strain, with the order of enzyme activity being three-copy > two-copy > five-copy > four-copy (Figure 3). Subsequent experiments utilized the three-copy strain.

Increasing the copy number provides additional opportunities for gene transcription and translation, leading to enhanced synthesis of the enzyme protein and higher total enzyme levels. Elevated enzyme concentrations increase the effective reaction rate. However, further increases in copy number may trigger copy number effects, including metabolic burden, protein aggregation, regulatory imbalance, and cellular overload, which can accelerate protein degradation and impair activity. Therefore, these factors must be considered to optimize protein expression and maintain enzymatic activity.

**Figure 4:**
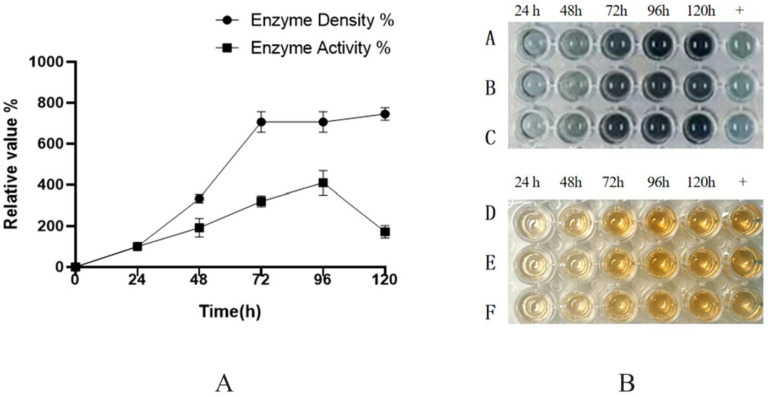
(**A**): Induced activity of GS115-BDM-HRP-C-3C strain at 120 h; Student’s *t*-test was used for statistical analysis, and a non-significant difference was detected between the control and experimental groups. (**B**): A, B, C are reactions with 5 μL enzyme solution added to 100 μL ABTS solution, reacted for 2 min, and then terminated with 2 M sulfuric acid; D, E, F are reactions with 5 μL enzyme solution added to 100 μL Guaiacol solution, reacted for 2 min, and then terminated with 2 M sulfuric acid.

Shake-flask experiments using the GS115-BDM-HRP-C-3C strain (Figure 4) demonstrated that both expression and activity peaked on the fourth day, followed by a decline on the fifth day. Over time, nutrient depletion, pH fluctuations, and metabolite accumulation likely inhibited cellular growth and metabolism, thereby reducing protein expression.

### 2.3. Western Blot (WB) and Glycosylation Analysis of HRP-C

HRP protein expressed in *Pichia pastoris* is highly glycosylated. EndoH was used to deglycosylate HRP protein from shake-flask fermentation.

**Figure 5:**
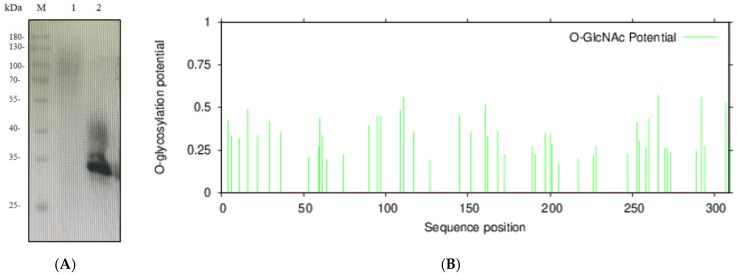
(**A**): Western Blot analysis of HRP-C protein expressed in *Pichia pastoris*. M: Protein Ladder; Lane 1: HRP-C fermentation supernatant; Lane 2: Deglycosylated HRP-C fermentation supernatant; (**B**): Prediction of HRP-C protein O-glycosylation sites.

As shown in Figure 5A, untreated protein exhibited significant smearing within the 40–180 kDa range, indicating extensive glycosylation. After EndoH treatment, a distinct band appeared at the target molecular weight, but smearing persisted between 35–45 kDa. This suggests that EndoH effectively removes N-glycosylation but has limited efficacy against O-glycosylation. O-glycosylation sites of HRP-C were predicted using an online tool (https://services.healthtech.dtu.dk, accessed on 14 October 2025), confirming the presence of both N- and O-glycosylation (Figure 5B).

**Figure 6:**
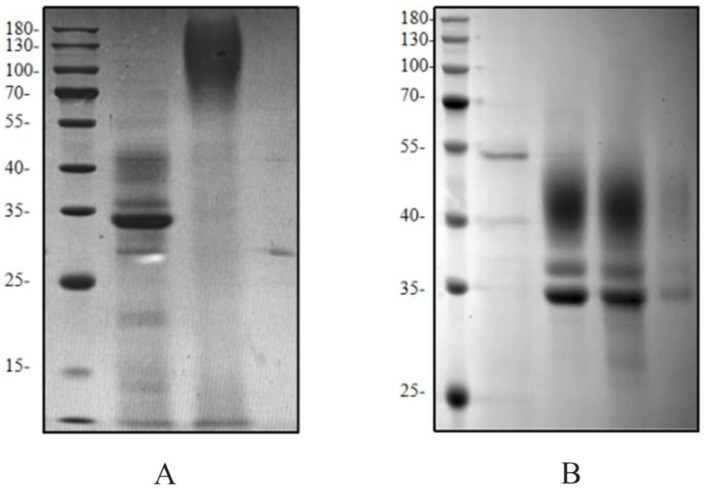
Purification analysis of HRP-C protein expressed in *Pichia pastoris*. (**A**): Purification of HRP-C fermentation supernatant: M: Protein Ladder; Lane 1: Deglycosylated HRP-C fermentation supernatant; Lane 2: Purified HRP-C fermentation supernatant; Lane 3: EndoH; (**B**): Purification of deglycosylated HRP-C fermentation supernatant: Lane 4: Flow-through; Lane 5: 100 mM imidazole elution; Lane 6: 200 mM imidazole elution; Lane 7: 500 mM imidazole elution.

During affinity purification of HRP-C fermentation supernatant, the target band was scarcely visible on SDS-PAGE (Figure 6). Analysis of the flow-through revealed poor binding to nickel beads. After thorough deglycosylation, most enzymes bound to the beads, improving purification efficiency. This indicates that glycosylation masks the His-tag, reducing its affinity for nickel ions and lowering purification efficiency. Glycosylation may also interfere with protein detection and antibody binding, affecting assay results.

### 2.4. Transcriptional Level Analysis of Multi-Copy HRP-C

**Figure 7:**
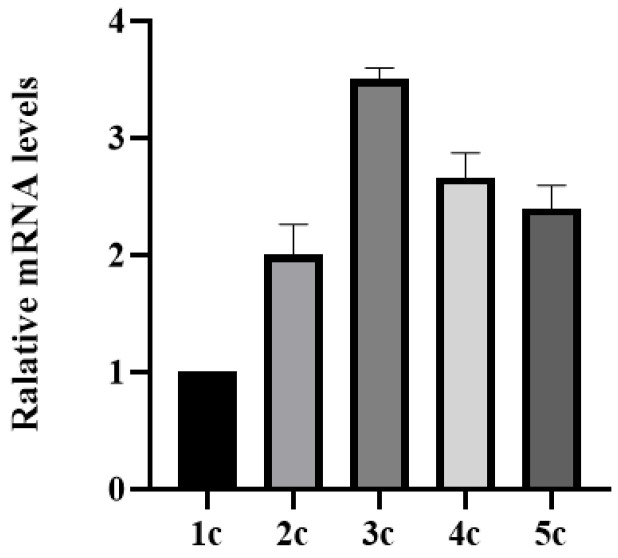
Transcription level analysis of yeast expression strains with 1–5 copies. Student’s *t*-test was used for statistical analysis, and a non-significant difference was detected between the control and experimental groups.

RNA was extracted from *Pichia pastoris* cells with 1–5 copies using an RNA extraction kit (Figure 7). qPCR analysis with triplicate replicates showed that transcriptional levels increased with copy numbers up to three copies but declined at four copies. This suggests that excessive copy numbers may impair transcriptional efficiency.

### 2.5. Co-Expression of Molecular Chaperones Enhances HRP-C-3C Expression in Pichia pastoris

**Figure 8:**
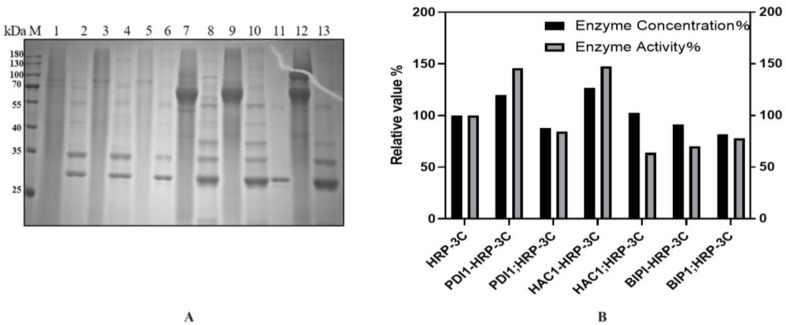
(**A**): SDS-PAGE analysis of BDM-HAC1-HRP-C shake flask fermentation. M: Protein Ladder; Lane 1–4: Results of BDM-HAC1-HRP-C shake flask fermentation; Lane 7–10: BDM-HAC1-HRP-C; Lane 11: Deglycosylation enzyme. (**B**): Relative activity analysis of HRP-C from co-expressed molecular chaperone strains in shake flask fermentation.

To further improve HRP expression, three molecular chaperones (PDI1, HAC1, BIP1) were co-expressed via two strategies:

1. Constitutive and Inducible Expression: The constitutive promoter PGAP drove chaperone expression, while the inducible promoter PAOX1 controlled HRP-C-3C. Gene sequences of yeast-derived chaperones were synthesized by Wuhan GenScript. PCR-amplified genes were cloned into the pGAPZa-A vector, followed by T5 exonuclease treatment and transformation. Recombinant plasmids (pGAPZa-A-PDI1, pGAPZa-A-HAC1, pGAPZa-A-BIP1) were electroporated into GS115. Competent cells were prepared and transformed with BDM-HRP-C-3C for induction.
2. Co-Induction on a Single Vector: Chaperones and HRP-C-3C were co-expressed on the same vector. Using the above plasmids as templates, chaperone genes were amplified and ligated into BDM-C-3C via T5 exonuclease. Recombinant plasmids (BDM-PDI1-HRP-C-3C, BDM-HAC1-HRP-C-3C, BDM-BIP1-HRP-C-3C) were verified by sequencing and transformed into GS115. Shake-flask experiments showed that BDM-PDI1-HRP-C-3C and BDM-HAC1-HRP-C-3C increased expression by 1.2- and 1.3-fold, respectively, with enzyme activity rising by nearly 1.5-fold. BDM-BIP1-HRP-C-3C showed no improvement (Figure 8B).

As shown in Figure 8B, the co-induction expression of molecular chaperones and HRP demonstrated better performance. This phenomenon might be attributed to the fact that pGAPZa is primarily induced by glycerol. During the cell accumulation phase, the co-expression of molecular chaperones may potentially compete with HRP-C for glycerol resources, leading to reduced biomass of HRP-C strains and consequently diminished subsequent expression levels. Moreover, while molecular chaperones may provide beneficial effects initially, their expression decline or degradation observed on the second day of induction could conversely introduce adverse effects, potentially leading to the accumulation of detrimental metabolic byproducts.

### 2.6. High-Density Fermentation of HRP-C

**Figure 9:**
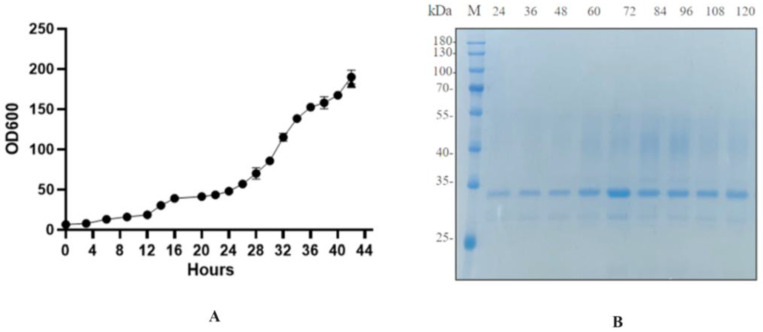
Analysis of 50 L high-hdensity fermentation of HRP-C. (**A**): Biomass growth during high-density fermentation; (**B**): SDS-PAGE of HRP-C from 50 L high-density fermentation (M: Protein Ladder; lanes correspond to fermentation times of 24 h, 36 h, 48 h, 60 h, 72 h, 84 h, 96 h, 108 h, and 120 h, with fermentation time starting from 24 h post-induction). Student’s *t*-test was used for statistical analysis, and a non-significant difference was detected between the control and experimental groups.

**Figure 10:**
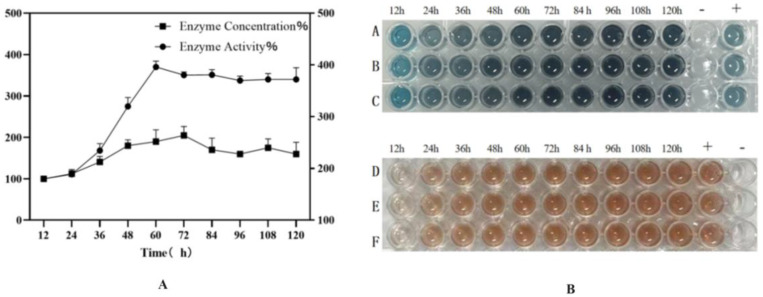
(**A**): Enzyme activity and protein concentration of high-density fermentation supernatant; (**B**): A, B, C represent the reaction of 5 µL enzyme solution added to 100 µL ABTS reaction solution for 2 min, with the reaction stopped by 2 M sulfuric acid; D, E, F represent the reaction of 5 µL enzyme solution added to 100 µL Guaiacol reaction solution for 2 min, with the reaction stopped by 2 M sulfuric acid. Student’s *t*-test was used for statistical analysis, and a non-significant difference was detected between the control and experimental groups.

The recombinant strain BDM-PDI1-HRP-C-3C was cultivated to high cell density in a 50-L bioreactor. During the initial glycerol batch phase, dissolved oxygen (DO) decreased rapidly due to high metabolic activity and was subsequently maintained at 20–30% through cascade control. The pH was controlled at 4.5 during the growth phase and shifted to 6.0 upon methanol induction to enhance heterologous protein expression. Methanol feeding was initiated at a rate of 2 mL/L/h for 6–8 h as an adaptive phase, followed by an increase to 7 mL/L/h; this controlled strategy prevented methanol accumulation and supported sustained protein production. Induction commenced when the OD_600_ reached 180–200 (Figure 9A). Samples collected every 12 h post-induction (up to 120 h) were treated with Endo H and analyzed by SDS-PAGE and activity assays (Figure 9B). The target protein concentration reached a maximum of 200 mg/L at 72 h, accompanied by an activity of 1796 U/mL, representing a ten-fold increase over shake-flask fermentation and the highest yield reported to date (Figure 10).

The successful scale-up to a 50-L bioreactor, achieving a ten-fold yield increase, validates the fermentation strategy. First, the peak productivity at 72 h (Figure 9A,B) followed by a plateau suggests potential limitations, such as protease degradation or product inhibition, which could be addressed in future studies by engineering protease-deficient strains or implementing continuous product removal. Second, the consistent activity across time points (Figure 10) indicates robust protein folding and secretion, likely benefiting from the PDI1 co-expression. Finally, the high activity in both ABTS and guaiacol assays (Figure 10B) confirms the biological relevance of the produced enzyme, supporting its potential for industrial applications. Further research could optimize the feeding strategy post-72 h to sustain the peak production phase.

### 2.7. Enzymatic Properties of Horseradish Peroxidase

**Figure 11:**
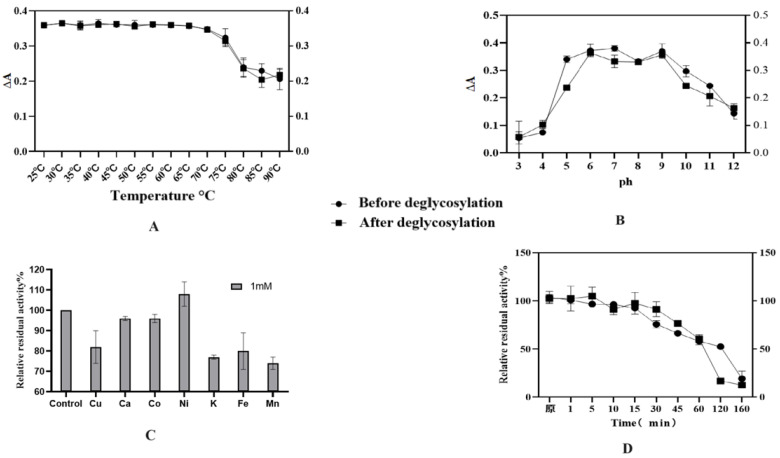
(**A**): The effect of temperature on HRP activity, ΔA(Absorbance 460); (**B**): The effect of pH on HRP activity; (**C**): The effect of metal ions on HRP activity; (**D**): Thermal stability study of HRP. Student’s *t*-test was used for statistical analysis, and a non-significant difference was detected between the control and experimental groups. Chinese character in subfigure (**D**) means “original”.

The enzymatic characterization results revealed that the optimal temperature for HRP-C was 37 °C, with the highest activity observed within the range of 30–45 °C (Figure 11A). Additionally, HRP-C exhibited thermotolerance, retaining biological activity at 60 °C, though its activity declined sharply above this temperature. No significant differences were observed between glycosylated and deglycosylated forms of the enzyme. Under varying pH conditions (Figure 11B), HRP activity showed distinct variations. The enzyme demonstrated high activity and stability between pH 6 and 9, with peak activity observed at pH 6 and 9, indicating favorable catalytic performance and stability in neutral to weakly alkaline environments. Furthermore, Ni^2+^ ions enhanced the enzyme’s activity, whereas other metal ions (Cu^2+^, Fe^3+^, K^+^, Mn^2+^) strongly inhibited its function (Figure 11C). Thermal stability assays demonstrated that HRP-C retained approximately 90% activity after incubation at 60 °C for 15 min, with activity beginning to decline after 30 min. The calculated half-life of the glycosylated enzyme was approximately 128 min, compared to 98 min for the deglycosylated form (Figure 11D).

The enzymatic characterization of HRP-C reveals robust catalytic performance under physiological conditions, with optimal activity at 37 °C and sustained thermotolerance up to 60 °C, consistent with its mesophilic nature and industrial applicability. The pH-dependent activity profile (peak at pH 6–9) aligns with HRP’s structural stability in neutral-alkaline environments, likely due to preserved heme accessibility and protonation states of critical residues. Notably, Ni^2+^-induced activation suggests metal coordination may modulate substrate binding, while inhibition by Cu^2+^/Fe^3+^ implies competitive heme interactions or oxidative damage. The marginal difference in thermal half-life between glycosylated and deglycosylated forms underscores that N-glycans contribute modestly to conformational stability, though catalytic core functionality remains intact. These findings support HRP’s versatility in biocatalytic applications, with minor glycosylation-dependent effects on operational stability.

**Figure 12:**
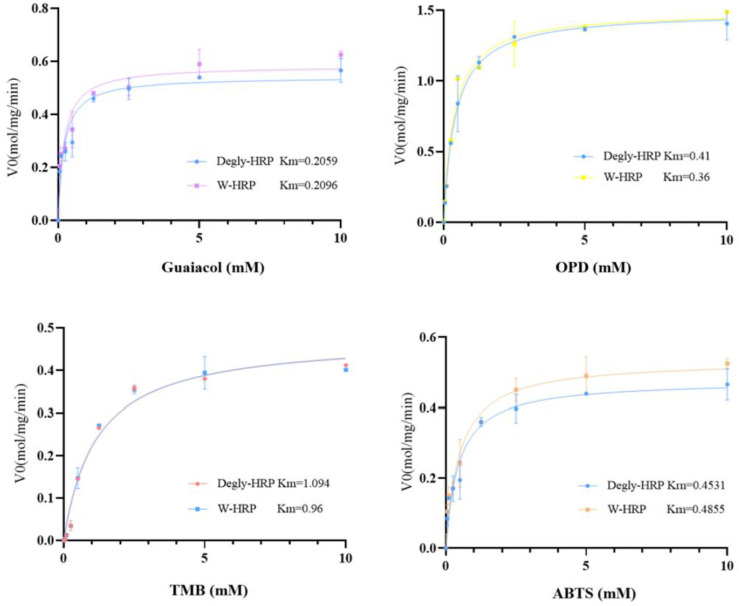
HRP enzyme kinetics constant curves. Student’s *t*-test was used for statistical analysis, and a non-significant difference was detected between the control and experimental groups.

In this experiment, four substrates—ABTS, TMB, OPD, and Guaiacol—were selected to investigate the enzymatic kinetic constants of HRP (horseradish peroxidase) before and after deglycosylation with different substrates. The determined enzymatic kinetic constants are shown in Table 1, and the fitted Michaelis-Menten curves are presented in Figure 12. The maximum reaction rates (V_max_) of HRP when fully saturated with the substrates, ranked from slowest to fastest, are as follows: Guaiacol, ABTS, TMB, and OPD. The affinity of HRP for the four substrates, ranked from highest to lowest, is: Guaiacol, OPD, ABTS, and TMB. At the same concentration, the catalytic efficiency of HRP for the different substrates, ranked from highest to lowest, is: OPD, Guaiacol, ABTS, and TMB. There were no significant differences in the maximum reaction rates, substrate affinity, or catalytic efficiency of HRP before and after deglycosylation with the different substrates.

The kinetic analysis reveals that HRP’s catalytic efficiency (kcat/Km) is primarily governed by substrate-specific properties rather than glycosylation status. OPD exhibited the highest efficiency, attributable to its favorable redox potential and steric compatibility with the active site, facilitating rapid electron transfer. Conversely, TMB’s lower efficiency may stem from steric hindrance or slower oxidation kinetics. Crucially, the negligible differences in Km, Vmax, and kcat between glycosylated and deglycosylated HRP across all substrates demonstrate that N-glycans are not essential for catalytic function or substrate binding. This suggests the enzyme’s active site architecture and electron transfer mechanism remain intact post-deglycosylation. The results indicate that for biotechnological applications requiring simplified production, glycosylation-minimized HRP variants could be employed without compromising kinetic performance with these common substrates.

## 3. Materials and Methods

### 3.1. Strains, Reagents, and Media

*Pichia pastoris*: GS115 and *E. coli* DH5α strains were purchased from Invitrogen (Carlsbad, CA, USA). The pHBM905BDM plasmid was constructed in our laboratory. Media: MD: 1.34% YNB, 2% glucose, 4 × 10^−5^% biotin; BMGY: 1% yeast extract, 2% peptone, 100 mM potassium phosphate, pH 6.0, 1.34% YNB, 4 × 10^−5^% biotin, 1% glycerol; BMMY: 1% yeast extract, 2% peptone, 100 mM potassium phosphate, pH 6.0, 1.34% YNB, 4 × 10^−5^% biotin; all prepared according to the *Pichia pastoris* expression manual (Invitrogen).

### 3.2. Plasmids and Primers

The plasmids and primers used in this study are listed in the following table, and the methods for plasmid construction are described in the subsequent sections.

### 3.3. Construction of pHBM905BDM-HRP-C Multi-Copy Expression Vectors

The HRP-C gene was optimized based on the codon usage preference of *Pichia pastoris* and synthesized by Sangon BioTech (Shanghai, China). The target gene was then amplified, and the pHBM905BDM vector was amplified to obtain the vector. The amplified fragments and vector were mixed, treated with T5 exonuclease, and transformed conventionally. Single colonies were selected for colony PCR screening, and the correct transformants were sent to Wuhan GenScript (Wuhan, China) for sequencing verification. After confirmation, the plasmid was named pHBM905BDM-HRP-C.

The multi-copy construction method was performed in vitro. *Xba*I and *Spe*I are a pair of isocaudomers. The plasmid was double-digested with *Xba*I and *BamH*I to obtain the fragment, and double-digested with *Spe*I and *BamH*I to obtain the vector. Taking the construction of 2 copies as an example, *Spe*I and *BamH*I were used to digest HRP-C to obtain the vector backbone. Meanwhile, *Xba*I and *BamH*I were used to digest the BDM-HRP-C expression cassette to obtain the cassette containing the target gene. After successful recovery and purification of these two bands, T4 DNA ligase was used for ligation, followed by conventional transformation. Multiple single colonies were selected from the transformation plate. Plasmids were extracted and verified by *Xba*I and *BamH*I digestion. Based on the gel electrophoresis results, plasmids matching the target bands were further verified by *Spe*I and *BamH*I, *SalI* digestion, thus obtaining the 2-copy expression plasmid. Similarly, 2-5 copy plasmids were successfully constructed, and after confirmation, the recombinant vectors were named pHBM905BDM-HRP-C-2C, pHBM905BDM-HRP-C-3C, pHBM905BDM-HRP-C-4C, and pHBM905BDM-HRP-C-5C.

### 3.4. Construction of Co-Expression Molecular Chaperone Expression Vectors

Co-induction plasmid construction: Using pGAPZa-A-PDI1, pGAPZa-A-HAC1, and pGAPZa-A-BIP1 plasmids (Table 2) as templates, the target genes were amplified using corresponding primers. Using BDM as a template, the corresponding vector was obtained by reverse amplification using corresponding primers (Table 3). The fragments and vectors were mixed, treated with T5 exonuclease, and transformed conventionally. The next day, multiple single colonies were randomly selected from the transformation plate, and colony PCR was performed using primers AOX-F and AOX-R (see Table 3), followed by agarose gel electrophoresis (using BDM-HAC1 as an example). Randomly selected transformants were sent to GenScript for sequencing verification. After successful verification, the recombinant plasmids were named as BDM-PDI1, BDM-HAC1, and BDM-BIP1. Subsequently, the molecular chaperones and BDM-HRP-C-3C were concatenated using isocaudomer ligation. After verification, the recombinant plasmids were named as BDM-PDI1-HRP-C-3C, BDM-HAC1-HRP-C-3C, and BDM-BIP1-HRP-C-3C.

Separate induction plasmid construction: The constitutive promoter P_GAP_ was used to express molecular chaperones, and the inducible promoter P_AOX1_ was used to express HRP-C-3C, which were sequentially inserted into the *Pichia pastoris* GS115 genome. The gene sequences of several commonly used yeast-derived molecular chaperones were obtained from NCBI and synthesized by Wuhan GenScript. The target genes were obtained by PCR using corresponding bidirectional primers, and the pGAPZa-A vector was obtained by reverse amplification using bidirectional primers. After T5 treatment and transformation, pGAPZa-A-PDI1, pGAPZa-A-HAC1, and pGAPZa-A-BIP1 plasmids were obtained.

### 3.5. Expression of HRP-C in Pichia pastoris

Taking pHBM905BDM-HRP-C as an example, pHBM905BDM-HRP-C was electroporated into GS115, and after screening for positive strains, single colonies were inoculated into BMGY medium and cultured in a shaker at 28–30 °C, 220 rpm. When the OD_600_ reached 6–8, the medium was replaced with BMMY medium. After glycerol was completely consumed, 1% methanol was added for induction which was performed every 12 h for 3–5 days. During the induction process, 4–10 μM hemin chloride was added. A 12% (*w*/*v*) SDS-PAGE was used to detect the recombinant proteins. Prepare a 12% separating gel by mixing 4.0 mL 30% acrylamide, 2.5 mL 1.5 M Tris-HCl (pH 8.8), 100 µL 10% SDS, 100 µL 10% APS, and 10 µL TEMED, then overlay with water for polymerization. After 30 min, prepare a 4% stacking gel and insert a comb. Mix protein samples 1:1 with 2× Laemmli buffer, boil at 95 °C for 5 min, and centrifuge. Load samples and run at 80 V (stacking gel) followed by 120 V (resolving gel) until the dye front reaches the bottom. Stain with Coomassie Blue R-250 (0.1% in 10% acetic acid/40% methanol) and destain until bands are visible.

### 3.6. High-Density Fermentation of Recombinant Strains and HRP Purification

First, seed culture was prepared by streaking on YPD plates, and single colonies were inoculated into YPD medium and cultured overnight at 28 °C. A 50 L fermenter was filled with 30 L BSM medium and sterilized thoroughly. 50 mL PTM1 basal salts were added, and the pH was adjusted to around 5.5. 3 L of seed culture was inoculated, and 50 mL PTM1 basal salts were added. After sterilization, glycerol was added, and after about 20 h, the glycerol was completely consumed. The DO value rapidly increased, and 50% glycerol was fed at intervals of 2 h. Samples were taken to measure the wet cell weight until the OD_600_ reached 180–200. When the pH suddenly increased, it indicated that the glycerol was completely consumed, and methanol was slowly fed at a rate of 2 mL/L/h after 6–8 h, followed by a rate of 7 mL/L/h. During the induction process, 10 μM hemin chloride was added.

The fermentation broth was centrifuged at 5000× *g* for 20 min at 4 °C to remove cellular debris, followed by filtration through a 0.45 μm membrane (Millipore, Burlington, MA, USA). The clarified supernatant was loaded onto a Ni-NTA affinity column (Qiagen, Beijing, China) pre-equilibrated with binding buffer (50 mM NaH_2_PO_4_, 300 mM NaCl, 10 mM imidazole, pH 8.0). After sample loading, the column was washed with 10 column volumes (CV) of wash buffer (20–50 mM imidazole in binding buffer) to remove nonspecifically bound proteins. The His-tagged HRP was eluted using 250 mM imidazole in binding buffer. Elution fractions were collected and analyzed by 12% SDS-PAGE under reducing conditions, followed by Coomassie Brilliant Blue R-250 staining to assess purity. Protein concentration was determined using the Bradford assay with BSA as standard. Fractions containing purified HRP were pooled and dialyzed against storage buffer to remove imidazole. The purified protein was aliquoted and stored at −80 °C until use.

### 3.7. Enzymatic Properties Analysis of HRP-C

The guaiacol method, based on the national standard GB/T 32131-2015 [19] for horseradish peroxidase (HRP) activity detection, measures enzyme activity spectrophotometrically at 470 nm. HRP catalyzes the oxidation of guaiacol by H_2_O_2_ to produce a brown product, with one enzyme unit defined as the amount converting 1 μmol H_2_O_2_ per minute at pH 7.0 and 25 °C. This method is widely adopted for its reproducibility and minimal reagent toxicity.

Optimum Temperature Determination: Reaction temperatures were set at 30 °C, 35 °C, 40 °C, 45 °C, 50 °C, 55 °C, 60 °C, 65 °C, 75 °C, and 85 °C. The deglycosylated enzyme, reaction buffer, and substrate were mixed and incubated for 2 min, and the reaction was terminated with acetic acid. Activity was measured, and each reaction was performed in triplicate.

Optimum pH Determination: Reaction buffers with pH values of 3, 4, 5, 6, 7, 8, 9, 10, 11, and 12 were prepared. After incubation at 4 °C for 12 h, the activity at different pH values was measured, and each reaction was performed in triplicate.

Thermal Stability Determination: The enzyme was incubated in a 60 °C water bath for 0, 1, 5, 10, 15, 30, 45, 60, 120, and 160 min, and the activity was measured. Each reaction was performed in triplicate.

Effect of Metal Ions on Enzyme Activity: The enzyme was added to solutions of metal ions (CaCl_2_, CoCl_2_, CuSO_4_, Fe_2_(SO_4_)_3_, KCl, LiCl, MgCl_2_, MnCl_2_, NiSO_4_, ZnCl_2_) with final concentrations of 0.5 and 1 mM, and the activity was measured. Each reaction was performed in triplicate.

Enzyme Kinetic Constant Determination: 20 μL of 0.01 mg/mL enzyme was mixed with the substrate and incubated at 37 °C for 5 min. The final substrate concentration was 0.05-10 mmol/L, and the reaction was initiated by adding 1 mM H_2_O_2_. The reaction system was 3 mL, and the absorbance was measured. The Michaelis-Menten equation was fitted using GraphPad Prism 10 software to calculate Km, Vm, and Kcat values.

## 4. Conclusions

Horseradish peroxidase (HRP) is an enzyme widely used in biochemical detection. However, its expression in prokaryotic systems such as *Escherichia coli* is challenging, primarily due to the complexity of its maturation and folding processes in non-native hosts [1,2,3,4,5]. HRP often forms inactive inclusion bodies, necessitating complex refolding procedures to potentially obtain active proteins. Additionally, the glycosylation of HRP, which is crucial for its activity, is difficult to achieve in prokaryotic expression systems [6,7,8,9]. In this study, we attempted to express HRP in *Pichia pastoris* and successfully obtained a high-yield recombinant *P. pastoris* strain through strategies such as gene dosage optimization, co-expression of molecular chaperones, and high-density fermentation.

This study retrieved information on various HRP isoenzymes from the NCBI database. First, based on literature review, proteins likely to be difficult to express were excluded, followed by comparative analysis of the obtained amino acid sequences. Subsequently, using protein structure simulation techniques, the protein with the largest heme pocket size and potentially the highest catalytic activity was selected, ultimately choosing HRP-C for further investigation.

The expression of HRP in the *P. pastoris* system involved two main aspects: First, the HRP gene sequence was optimized according to the codon preference of *P. pastoris*. The optimized gene was cloned into the BDM expression vector and then transformed into *P. pastoris* GS115. After screening, a high-expression, high-activity BDM-HRP-C strain was obtained. On this basis, multi-copy plasmids 2C–5C of BDM-HRP-C were successfully constructed and transformed into *P. pastoris* GS115 for expression. Experimental results showed that multi-copy significantly enhanced the expression level and functional activity of HRP. Among them, the three-copy recombinant strain BDM-HRP-C-3C achieved the highest target protein expression and activity, with a yield of 70 mg/L and an enzyme activity of 557 U/mg. The increase in gene copy number provided additional opportunities for gene transcription and translation, promoting the synthesis of more target proteins and increasing the total enzyme amount. The increase in protein quantity enhanced the effective concentration of the enzyme in reactions, thereby improving the reaction rate. However, as the gene copy number further increased to four or five copies, protein expression and activity showed a declining trend, likely due to the negative impacts of increased copy number on host cells, such as increased metabolic burden, protein aggregation, and regulatory imbalance, all of which may prevent further enhancement of protein expression. Second, in addition to multi-copy, molecular chaperones were introduced to further improve HRP expression. Different molecular chaperones (PDI1, HAC1, BIP1) were selected and co-expressed with BDM-HRP-C-3C using both co-induction and separate induction methods. The results showed that the yields of strains BDM-PDI1-HRP-C-3C and BDM-HAC1-HRP-C-3C increased by 1.4-fold, with enzyme activities increasing by 1.2-fold and 1.3-fold, respectively. Although the introduction of molecular chaperones in co-expression with HRP improved expression levels, the enhancement was limited. Further optimization of molecular chaperone expression levels or exploration of other regulatory approaches is needed to develop effective strategies and optimize the protein expression system.

In summary, this study integrated multiple strategies, including gene screening, expression system selection, gene dosage optimization, introduction of molecular chaperones, and high-density fermentation, ultimately obtaining a high-expression recombinant P. pastoris strain, pHBM905BDM-HRP-C-3C. After fermentation in a bioreactor, the protein expression level reached a maximum of 200 mg/L, with an enzyme activity of 1796 U/mL, the highest reported in the literature to date. This provides an efficient and sustainable method for producing specific enzymes in scientific research, offering technical support for related enzyme studies and applications. In the industrial sector, this achievement can supply high-quality, high-efficiency HRP products for biotechnology, medicine, and environmental protection, contributing to the development and innovation of related industries.

## Figures and Tables

**Table 1:** Kinetic Constants of HRP Enzyme.

Substrate		ABTS	TMB	OPD	Guaiacol
Λ (nm)		415	450	492	436
ε (M^−1^ cm^−1^)		36,000	39,000	16,700	25,500
Km (M)	Degly-HRP	4.535 × 10^−5^	9.60 × 10^−5^	4.10 × 10^−5^	2.059 × 10^−5^
	W-HRP	4.855 × 10^−5^	1.096 × 10^−5^	3.60 × 10^−5^	2.096 × 10^−5^
Vmax (M s^−1^)	Degly-HRP	4.761 × 10^−5^	4.76 × 10^−5^	1.490 × 10^−5^	5.413 × 10^−5^
	W-HRP	5.339 × 10^−5^	4.789 × 10^−5^	1.494 × 10^−5^	5.825 × 10^−5^
Kcat (s^−1^)	Degly-HRP	238	238	745	270
	W-HRP	267	239	747	291

**Table 2:** The plasmids used in the experiment.

Plasmids	Characteristics	Source
pHBM905BDM-HRP**-C**	AOX1 promoter, multicopy design, His4 selection, Ampr, pBR322 ori; Contains MF4I signal peptide and Cpo I/Not I cloning sites.	Made in our lab
pHBM905BDM-HRP-C-2C	AOX1 promoter, multicopy design, His4 selection, Ampr, pBR322 ori; Contains MF4I signal peptide and Cpo I/Not I cloning sites.	Made in our lab
pHBM905BDM-HRP-C-**3**C	AOX1 promoter, multicopy design, His4 selection, Ampr, pBR322 ori; Contains MF4I signal peptide and Cpo I/Not I cloning sites.	Made in our lab
pHBM905BDM-HRP-C-**4**C	AOX1 promoter, multicopy design, His4 selection, Ampr, pBR322 ori; Contains MF4I signal peptide and Cpo I/Not I cloning sites.	Made in our lab
pHBM905BDM-HRP-C-**5**C	AOX1 promoter, multicopy design, His4 selection, Ampr, pBR322 ori; Contains MF4I signal peptide and Cpo I/Not I cloning sites.	Made in our lab
pHBM905BDM-PDI1	AOX1 promoter, multicopy design, His4 selection, Ampr, pBR322 ori; Contains MF4I signal peptide and Cpo I/Not I cloning sites.	Made in our lab
pHBM905BDM-HACI	AOX1 promoter, multicopy design, His4 selection, Ampr, pBR322 ori; Contains MF4I signal peptide and Cpo I/Not I cloning sites.	Made in our lab
pHBM905BDM-BIP1	AOX1 promoter, multicopy design, His4 selection, Ampr, pBR322 ori; Contains MF4I signal peptide and Cpo I/Not I cloning sites.	Made in our lab
pHBM905BDM-HRP-C-3C-HACI	AOX1 promoter, multicopy design, His4 selection, Ampr, pBR322 ori; Contains MF4I signal peptide and Cpo I/Not I cloning sites.	Made in our lab
pHBM905BDM-HRP-C-3C-PDI1	AOX1 promoter, multicopy design, His4 selection, Ampr, pBR322 ori; Contains MF4I signal peptide and Cpo I/Not I cloning sites.	Made in our lab
pHBM905BDM-HRP-C-3C-BIP1	AOX1 promoter, multicopy design, His4 selection, Ampr, pBR322 ori; Contains MF4I signal peptide and Cpo I/Not I cloning sites.	Made in our lab
pGAPZa-A-PDI1	GAP promoter, pUC Ori, Zeocinr, alpha-factor signal, Xho I/Xba I cloning sites	Made in our lab
pGAPZa-A-HACI	GAP promoter, pUC Ori, Zeocinr, alpha-factor signal, Xho I/Xba I cloning sites	Made in our lab
pGAPZa-A-BIP1	GAP promoter, pUC Ori, Zeocinr, alpha-factor signal, Xho I/Xba I cloning sites	Made in our lab

**Table 3:** The primers used in the experiment.

Primers	Sequence (5’-3’)
FK-S-F	GGAGGTGGATCTTCTATGCAACTTACCCCAACCTTTTACGAC
FK-S-R	TTCTGAGTCCGACATTGACCGGGATCTTAATTCTACTTCCAAAGA
BDM-F	GAGGGGCGGCCGCGAATTAATTC
BDM-R	GGGATCGGACCGGGATCTTAATTCTACTTCC
BDM-G-F	GGGGCGGCCGCGAATTA
BDM-G-R	GGGATCGGACCGGGATCTTAATTC
BK-F	ATCCAGAAACAAATTAGAAAACTACGC
BK-R	AATTTGTTTCTGGATTCAACCTTGG

## Data Availability

The original contributions presented in this study are included in the article. Further inquiries can be directed to the corresponding authors.

## References

[1] Veitch N.C. (2004). Horseradish peroxidase: A modern view of a classic enzyme. Phytochemistry.

[2] Bobrow M.N., Harris T.D., Shaughnessy K.J., Litt H. (1989). Catalyzed reporter deposition, a novel method for signal amplification in immunological assays. J. Immunol. Methods.

[3] Näätsaari M., Krainer E., Glieder A. (2015). An updated view on horseradish peroxidases: Recombinant production and biotechnological applications. Appl. Microbiol. Biotechnol..

[4] Mirković N., Zihrul M., Žižić M., Marinković A., Prodanović R., Todorović T.R. (2022). The Influence of Isoenzyme Composition and Chemical Modification on Horseradish Peroxidase@ZIF-8 Biocomposite Performance. Polymers.

[5] Hartmann C., Montellano P.R.O.D. (1992). Baculovirus expression and characterization of catalytically active horseradish peroxidase. Arch. Biochem. Biophys..

[6] Segura M.D.L.M., Levin G., Miranda M.V., Santana M. (2005). High-level expression and purification of recombinant horseradish peroxidase isozyme C in SF-9 insect cell culture. Process Biochem..

[7] Kawaoka A., Kawamoto T., Moriki H., Takeuchi Y., Ito Y. (1994). Growth stimulation of tobacco plant introduced the horseradish peroxidase gene prxC1a. J. Ferment. Bioeng..

[8] John C.D., Andrew O.S. (1989). DNA Sequence Coding for HRP Enzyme. Patent Application.

[9] Smith A.T.S.N., Santama N., Dacey S., Ortiz P.A. (1990). Expression of a synthetic gene for horseradish peroxidase C in *Escherichia coli* and folding and activation of the recombinant enzyme with Ca^2+^ and heme. J. Biol. Chem..

[10] Morawski B., Lin Z., Fau-Cirino P., Cirino P.F., Joo H., Dean D.A. (2006). Functional expression of horseradish peroxidase in *Saccharomyces cerevisiae* and *Pichia pastoris*. Protein Expr. Purif..

[11] Shao Y., Xue C., Liu W., Wang L., Zhang B., Chen K. (2022). High-level secretory production of leghemoglobin in *Pichia pastoris* through enhanced globin expression and heme biosynthesis. Bioresour. Technol..

[12] Di Rocco G., Baldari S., Gentile A., Toietta G., Picozzi P., Magrini A., Ciliberto G., Aurisicchio L. (2018). Protein Disulfide Isomerase as a Prosurvival Factor in Cell Therapy for Muscular and Vascular Diseases. Stem Cell Res. Ther..

[13] Guerfal M., Ryckaert S., Jacobs P., Ameloot P., Thevelein J.M. (2011). The *HAC1* gene from *Pichia pastoris*: Characterization and effect of its overexpression on the production of secreted, surface displayed and membrane proteins. Microb. Cell Fact..

[14] Kranz P., Sänger C., Wolf A., Beschorner U., Moepps B., Laechelt S., Bruckner T., Grosse J., Bischoff E., Azoitei A. (2020). Tumor Cells Rely on the Thiol Oxidoreductase PDI for PERK Signaling in Order to Survive ER Stress. Sci. Rep..

[15] Yang S., Jackson C., Karapetyan E., Dutta P., Kermah D., Wu Y., Wu Y., Schloss J., Vadgama J.V. (2022). Roles of Protein Disulfide Isomerase in Breast Cancer. Cancers.

[16] Nielsen K.L., Indiani C., Henriksen A., Feis A., Becucci M., Gajhede M., Smulevich G., Welinder K.G. (2001). Differential Activity and Structure of Highly Similar Peroxidases. Spectroscopic, Crystallographic, and Enzymatic Analyses of Lignifying Arabidopsis thaliana Peroxidase A2 and Horseradish Peroxidase A2. Biochemistry.

[17] Ostergaard L., Teilum K., Mirza O., Mattsson O., Petersen M., Welinder K.G., Mundy J., Gajhede M., Henriksen A. (2000). Arabidopsis ATP A2 peroxidase. Expression and High-Resolution Structure of a Plant Peroxidase with Implications for Lignification. Plant Mol. Biol..

[18] Bernardes A., Textor L.C., Santos J.C., Cuadrado N.H., Kostetsky E.Y., Roig M.G., Bavro V.N., Muniz J.R., Shnyrov V.L., Polikarpov I. (2015). Crystal Structure Analysis of Peroxidase from the Palm Tree *Chamaerops excelsa*. Biochimie.

[19] (2015). Standard Operating Procedure for Determination of Horseradish Peroxidase Activity by Colorimetric Method.

